# Supplementary material for: Association between oral microbiome and seven types of cancers in East Asian population: a two-sample Mendelian randomization analysis
Source: Front Mol Biosci. 2023 Nov 21;10:1327893. doi: 10.3389/fmolb.2023.1327893 (PMC10702768; doi:10.3389/fmolb.2023.1327893)
Supplement: Supplementary file 1 [file DataSheet1.ZIP › Supplementary Materials/MR plots for tongue/tongue═╝/Lung cancer/pheno.2207_to_lung cancer_forest.pdf]

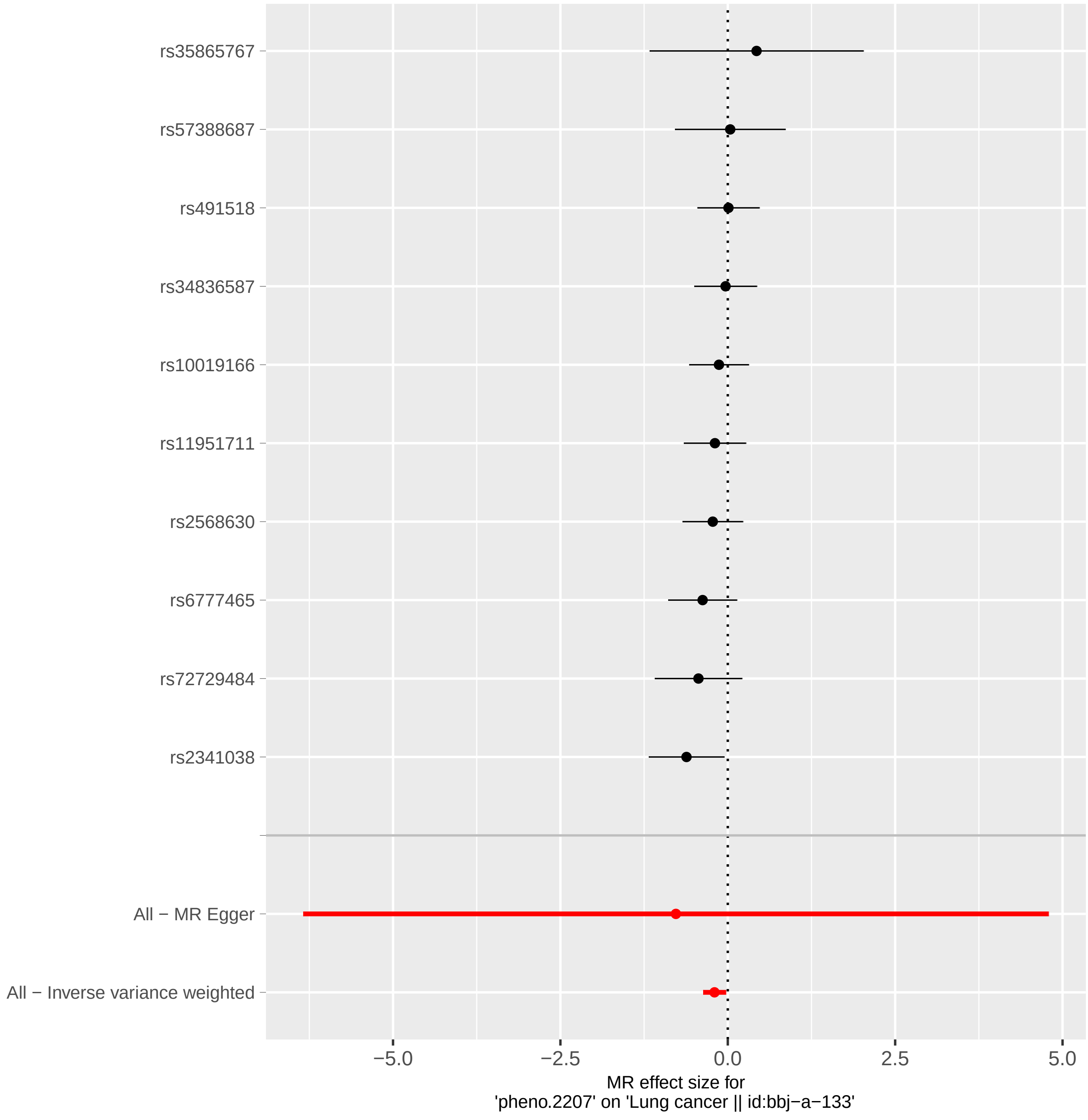
rs35865767
rs57388687
rs491518
rs34836587
rs10019166
rs11951711
rs2568630
rs6777465
rs72729484
rs2341038
All − MR Egger
All − Inverse variance weighted
−5.0
−2.5
0.0
2.5
5.0
MR effect size for
'pheno.2207' on 'Lung cancer || id:bbj−a−133'
